# A clitoral verrucous carcinoma in an area of lichen planus has aggressive features

**DOI:** 10.1186/s12957-016-1069-0

**Published:** 2017-01-06

**Authors:** Wiebren A. A. Tjalma, Vasiliki Siozopoulou, Manon T. Huizing

**Affiliations:** Department of Obstetrics and Gynecology, Multidisciplinary Breast Clinic, Gynecological Oncology Unit, Antwerp University Hospital - University of Antwerp, Wilrijkstraat 10, Edegem, 2650 Belgium

**Keywords:** Verrucous, Carcinoma, Lichen planus, Clitoris, Vulval, Treatment

## Abstract

**Background:**

Verrucous carcinoma of the vulva is extremely rare. It is a slow growing, low malignant variant of a squamous cell carcinoma with a cauliflower appearance. Women with lichen planus have an increased risk of developing vulval cancer.

**Case presentation:**

A 79-year-old woman consulted for vulval itching. On clinical examination, a 3-cm large verrucous clitoral cancer in an area of lichen planus was seen. Based on her last clinical examination, the growth was estimated to be 1 cm^2^ per month with an invasion depth after 6 months of 5 mm. A tumor developing in an area of lichen planus appears to have more aggressive features. This is the first time that the growth of a verrucous carcinoma has been documented in an area of lichen planus.

**Conclusions:**

Clinicians and patients should be aware of the aggressive behavior of cancers developing in areas of lichen planus and adjust their surgical management together with the follow-up strategy.

## Background

Vulval cancer is a rare disease. It encompasses 0.58% of all female cancers and 0.36% of all cancer deaths in women [1]. Eighty to 90% of all primary vulval cancers are of squamous origin. A verrucous carcinoma is a very rare variant of a squamous carcinoma. It was described for the first time in 1948 in the oral cavity [2]. Later on, also other locations were described like the cervix, vagina, bladder, penis, and scrotum [3].

A verrucous carcinoma is a distinct entity with an exo- and endophytic growth pattern, minimal cell atypia, and pushing margins in contrast to the invasive character of well-differentiated squamous carcinoma [2]. Macroscopically, the tumor has an aggressive cauliflower appearance, which can easily be confused with a Buschke-Löwenstein tumor [4]. Microscopically on the other hand it is a well-differentiated tumor with a low malignant potential. To the best of our knowledge, this is the first description of a verrucous carcinoma in an area of lichen planus.

## Case presentation

A 79-year-old woman consulted for vulval itching. Her past medical history included a breast cancer 2 years earlier. Her breast cancer was staged as pT1pN0M0 and treated by surgery and adjuvant tamoxifen 20 mg daily.

**Fig. 1:**
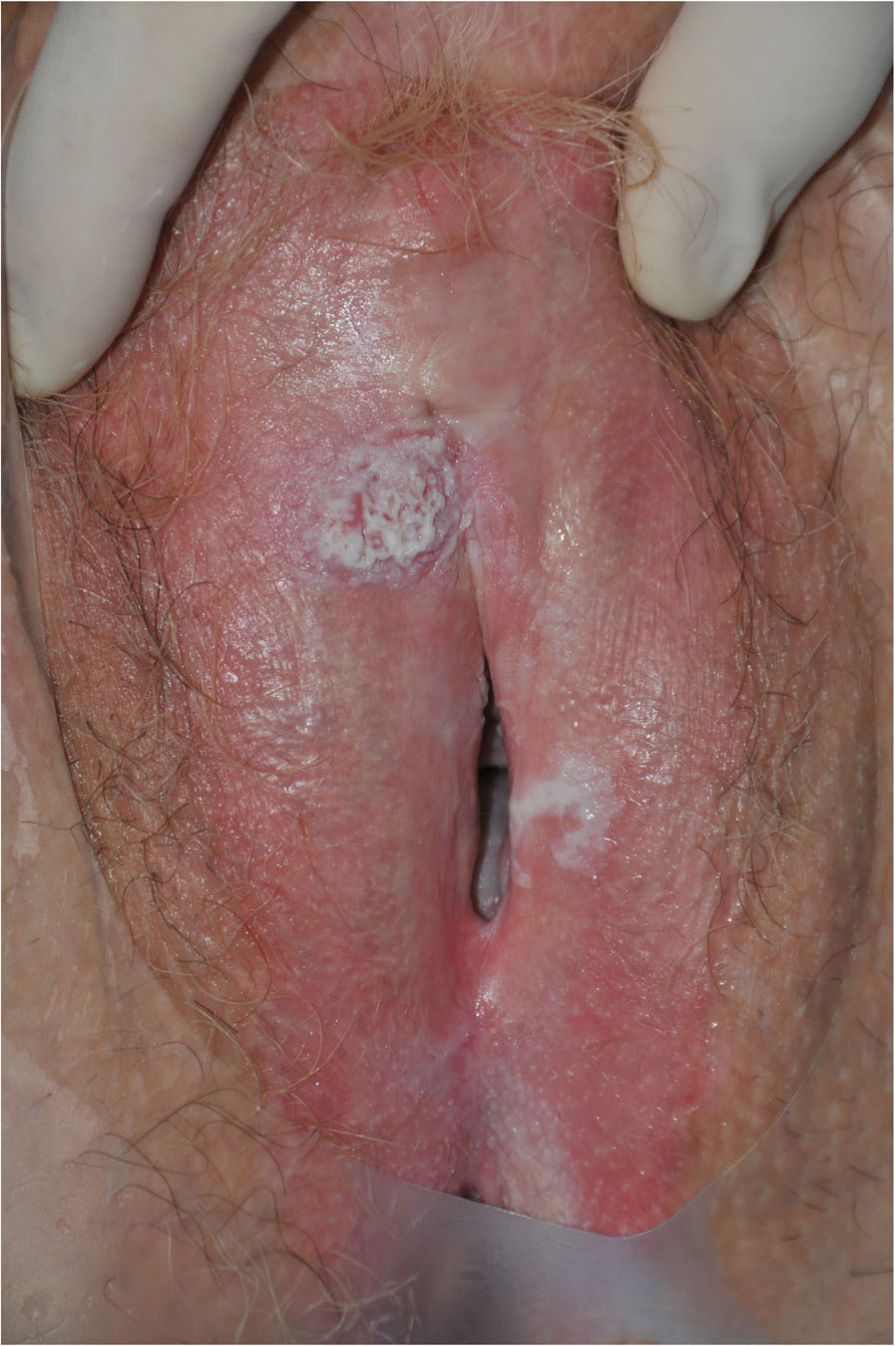
A grey-white area of lichen planus on the right sight of the vulva

On clinical examination, a pink vulva was seen with on the right side a grey-white area (Fig. 1). A biopsy was performed of this area and showed lichen planus. Due to the intense itching, the grey-white area was removed. She had a regular follow-up. She revisited the clinic 12 months later when the itching had recurred. The itching started 6 months earlier. Initially it did not bother her, but after a while she noted that a grey-white area was growing on her clitoris.

**Fig. 2:**
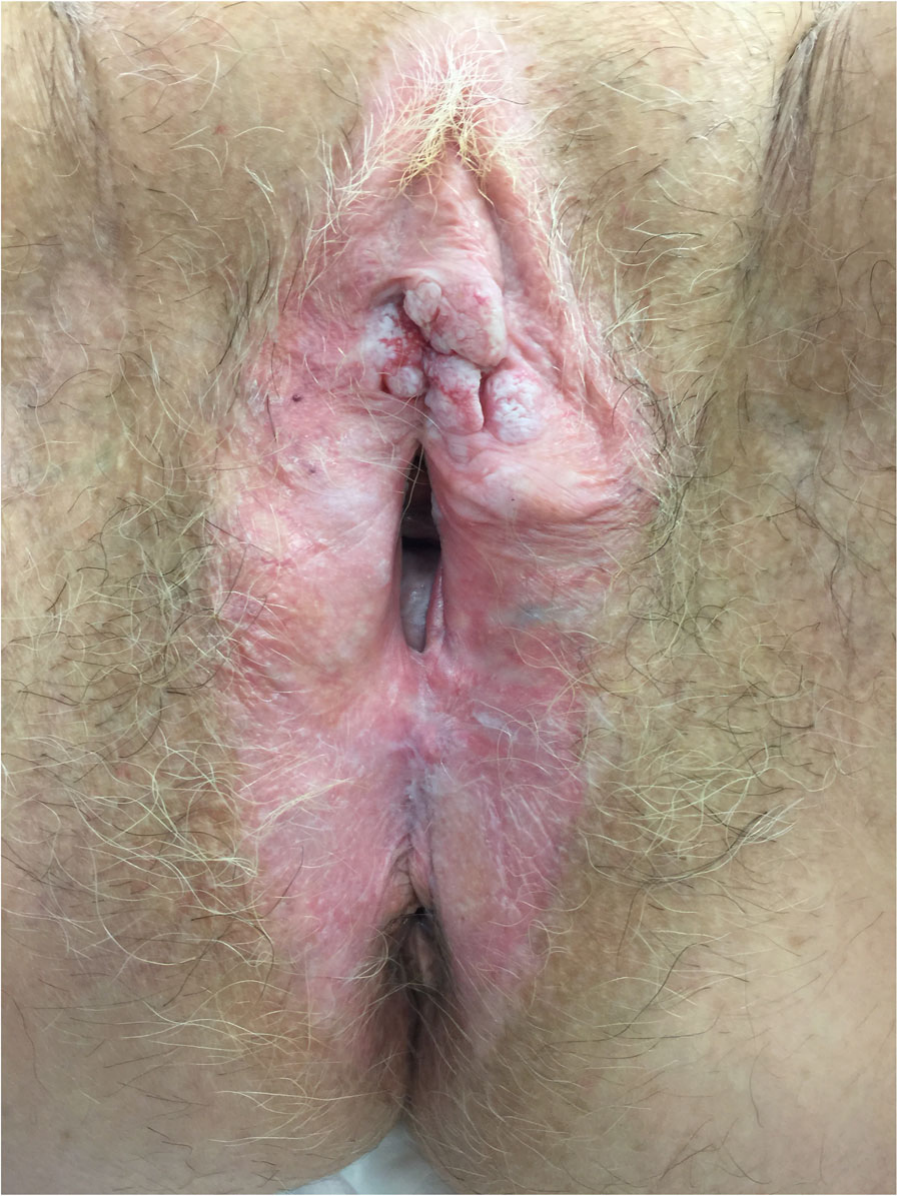
An exophytic and cauliflower-looking mass centrally on the clitoris

On clinical examination, an exophytic cauliflower-like mass of 3 cm in diameter was noted on the clitoris. The mass extended to the labia minus on both sites. The urethral meatus was free by a macroscopically margin of 5 mm. No groin nodes were palpated. On inspection, there was no involvement of the vagina or the cervix. Clinically, the mass had the appearance of a vulval cancer or a wart (Fig. 2). Due to the location of the tumor and its macroscopical features, it was opted to remove the tumor with a margin of 1 cm instead of performing a biopsy first.

**Fig. 3:**
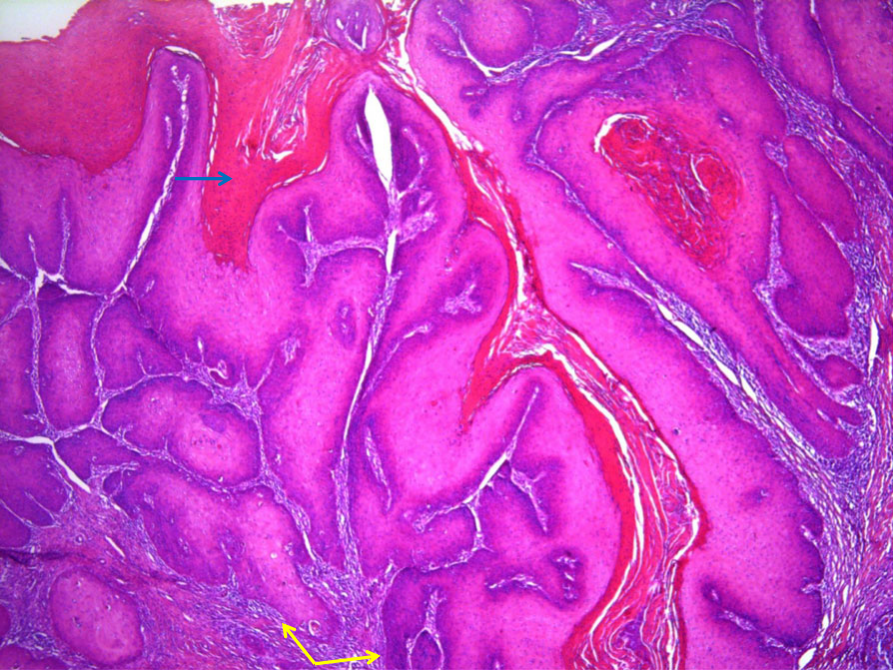
An endophytic lesion with hyper- and parakeratosis (*blue arrow*). There is no marked cytologic atypia. It invades deep into the dermis in the form of bulbous ends with a pushing border (*yellow arrows*) (hematoxylin-eosin staining, magnification ×40)

**Fig. 4:**
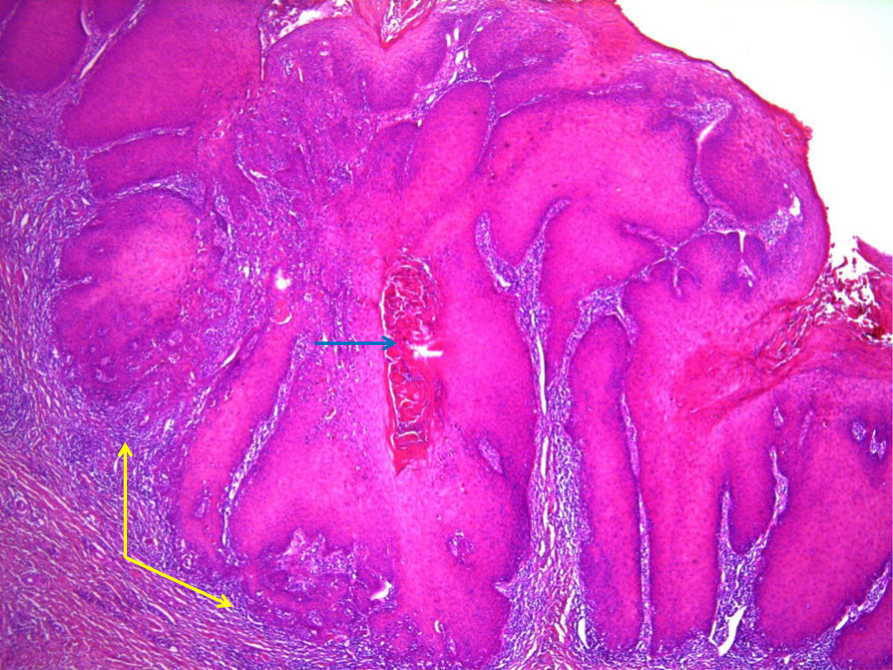
An endophytic lesion with hyper- and parakeratosis (*blue arrow*). There is no marked cytologic atypia. It invades deep into the dermis in the form of bulbous ends with a pushing border (*yellow arrows*) (hematoxylin-eosin staining, magnification ×40)

**Fig. 5:**
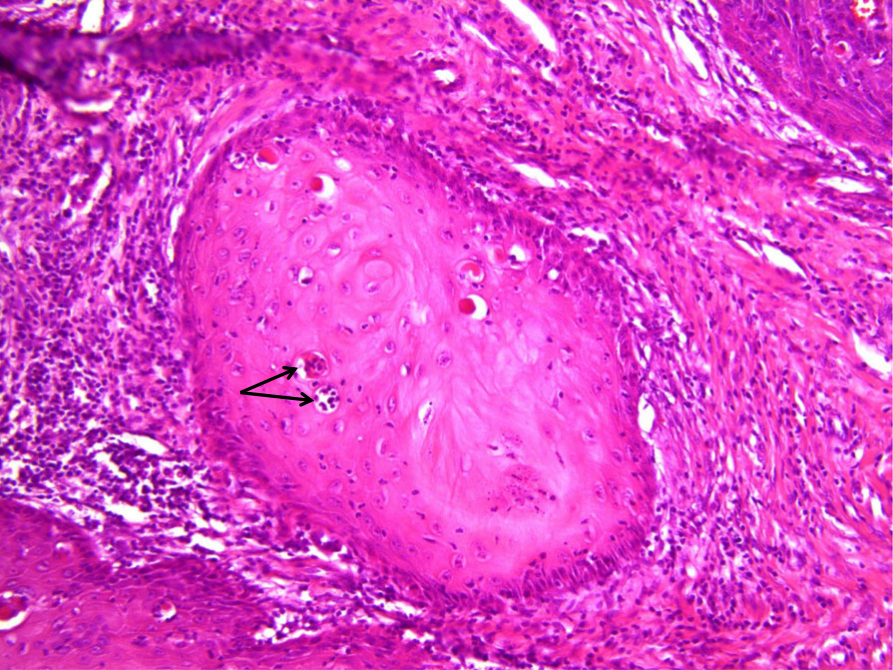
A highly differentiated tumor without marked atypia is seen. The cells resemble to normal epithelium. A characteristic feature is the infiltration of the tumor nests through neutrophils (*black arrows*) (hematoxylin-eosin staining, magnification ×40)

**Fig. 6:**
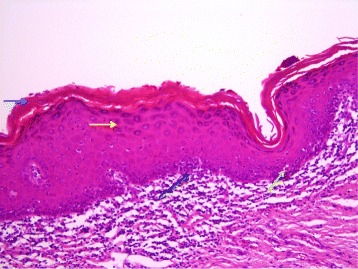
The adjacent epithelium shows features of lichen planus; hyperkeratosis without parakeratosis (*blue arrow*), hypergranulosis (*yellow arrow*), band-like inflammatory infiltrate in the underlying dermis with invasion into the basal layer of the epithelium (*black arrow*), vacuolation of basal keratinocytes and apoptotic cells (*green arrow*) (hematoxylin-eosin staining, magnification ×200)

Pathological examination showed an endophytic lesion with hyper- and parakeratosis (blue arrow) with only mild atypia (Figs. 3 and 4). The lesion invades deep into the dermis in the form of bulbous ends with a pushing border (yellow arrows) (Figs. 3 and 4). The tumor itself is a highly differentiated tumor with only mild atypia (Fig. 5). A characteristic feature is the infiltration of the tumor nests through neutrophils (black arrows) (Fig. 5). The adjacent epithelium shows features of lichen planus; hyperkeratosis without parakeratosis (blue arrow), hypergranulosis (yellow arrow), band-like inflammatory infiltrate in the underlying dermis with invasion of lymphocytes into the basal layer of the epithelium (black arrow), vacuolation of basal keratinocytes and apoptotic cells (green arrow) (Fig. 6). The HPV DNA assay revealed that the tumor was HPV negative. The definitive pathology showed a 2 × 3 cm well-differentiated verrucous carcinoma with tumor free margins. The margin to the urethra was 7 mm and all other margins were more than 1 cm. The maximum depth of infiltration was 5 mm.

Clinically, there rose a problem. Normally, the therapy would stop here; these verrucous cancers are slow growing and hardly metastasize. However, in our case, the tumor developed with a growth of 1 cm^2^ per month and had an infiltration depth of 5 mm after 6 months. These features gave the tumor an “aggressive”-looking behavior. For a verrucous carcinoma, no additional surgery would be performed. For a classic squamous carcinoma, one would perform a sentinel node biopsy and in case of a metastatic node a bilateral femoral inguinal node resection. In the current case, due to the aggressive features, there were three possible treatment options: (1) no groin surgery; (2) the classical bilateral inguinofemoral resection en bloc; (3) sentinel node biopsy and only in case of an involved lymph node an en bloc resection. Of course, there was always the possibility that due to the previous surgery no sentinel node could be identified. Together with the patient and her family (shared decision making), it was decided to perform a sentinel node procedure and if there were tumor cells in the sentinel node, an en bloc resection of both groins would be performed. In case no sentinel node could be identified in both groins, no groin surgery would be performed. In a second procedure, both sentinel nodes were identified with technetium. The preoperative examination of the nodes as well as the definitive results showed no tumor involvement of the lymph nodes. The tumor was staged as FIGO IB and no additional treatment was advised. At present, 29 months after the diagnosis, the patient is well with no signs of recurrence.

### Discussion

Verrucous carcinoma of the clitoris is extremely rare with only one previously described case in the literature [5]. A HPV infection can be detected in about one third of the cases [2, 3]. This seems consistent with the role of HPV in the development of vulval cancer in general.

Vulval cancers can be divided in two groups. The first group is the young patients with multicenter tumors associated with HPV infection, smoking, and precancerous lesions (vulvar intraepithelial lesions (VIN)). The other group is the older patients with HPV-negative tumors associated with lichen sclerosus and squamous hyperplasia [6]. It is estimated that about 5% of all VIN 3 lesions will progress to an invasive disease [7] and almost 7% of all squamous cancers of the vulva occur in an area of lichen sclerosus [8]. In the current case, the verrucous cancer developed in an area of lichen planus. In contrast to squamous cell carcinoma, the development of a verrucous carcinoma in cutaneous or oral lichen planus is rare. The first association of vulvar lichen planus and squamous cell cancer was reported in 1989 [9].

Lichen planus is a chronic inflammatory autoimmune dermatosis. In oral and esophageal mucosa, lichen planus has a cancer risk of 5% [10]. In HPV-negative penile cancer, lichen planus is recognized as a risk factor; but in HPV negative vulval cancer, this is surprisingly not the case [10–12]. HPV-negative squamous cell cancers associated with lichen planus have been located in the nonhair-bearing vulvar mucosa in 47% and in the vestibulum in 53% [10]. In the present case, the tumor was located on the clitoris at a microscopic distance of 7 mm from the urethra.

For lichen sclerosus or lichen planus without carcinoma, topical corticosteroids are the first choice. A second-line treatment is tacrolimus ointment. In case of a squamous carcinoma, surgery is the preferred option. An alternative option for a patient with a squamous cell carcinoma in a specific area can be photodynamic therapy.

The advised therapy however of a verrucous tumor is a wide local excision with a 1-cm tumor-free margin. Inadequate surgery will lead to an increased risk of recurrences. Primary radiotherapy is contraindicated as it has 11–30% risk of anaplastic transformation [2]. Verrucous carcinomas rarely metastasize to the inguinofemoral lymph nodes. Lichen planus-associated squamous carcinomas on the other hand are aggressive malignancies with inguinal metastases in 42% and a local recurrence after surgery of 39% [10]. Eighty-six percent of the recurrences occurred within 1 year and 37% died [10].

Verrucous carcinomas in general are regarded as slow growing tumors with an indolent character. In the current case and for the first time in literature, it was documented that there was a growth of 1 cm^2^ per month. In verrucous carcinoma, there is mostly only a minimal invasion of the superficial layers. The invasion depth in present case was 5 mm, and it was estimated that the tumor invaded 0.8 mm per month. The speed of the growth and the depth of the invasion are features of aggressiveness. The aggressive behavior of a verrucous carcinoma could be explained by the development of the tumor in an area of lichen planus. The progression and prognosis of a verrucous squamous cell carcinoma in the context of a lichen planus require further investigations.

Verrucous carcinomas have a high risk of local recurrence (30%). It is likely that in our case the patient will have a recurrence. The question is if you should call it a recurrence or the development of a new cancer in an area of lichen planus. Based on the fact that lichen planus is a risk factor, you better call it the development of a new cancer. Women with active lichen planus and also lichen sclerosis should be regarded as at risk for malignant transformation. A reduction in cancer progression could theoretically be induced by the treatment of active lichen planus or lichen sclerosis. A recent randomized controlled trial demonstrated improvements in the disease course of lichen planus with initial aggressive therapy involving oral prednisolone, with or without additional topical corticosteroids application, and maintenance therapy with weekly low-dose methotrexate when topical therapy was not adequate [13, 14]. The use of immunosuppressive agent is also often mentioned in the literature; however, they bear the risk of increased malignant transformation.

## Conclusions

Lichen planus has a chronic nature and there is often a delay in diagnosis of a verrucous cancer of several months. A verrucous carcinoma developing in an area of lichen planus has more aggressive features. Women should be informed or rather educated about the risk of malignant transformation in case of active lichen planus. If there are complaints, patients should use a topical corticosteroid. If there is no improvement within 5 days, they should contact their physician. Physicians should also be aware of the malignant potential of lichen planus and adjust their management accordingly.
